# Majority of scholarly journals from India in WoS and SCOPUS are closed access and promoted by foreign publishers: A 2026 concern on technological embargo

**DOI:** 10.6026/973206300222502

**Published:** 2026-02-28

**Authors:** Yogesh Singh Bisht, Ruby Singh, Senkuttuvan Pavithra, Sivakumar Monisha, Muthukumaran Kameshwari, Muthukumaran Kamaleshwari, Thangamani Yogasri, Anandhan Nisha, Marimuthu Vishnu Priya, Anandhan Viji

**Affiliations:** 1 AskBisht (Academic Publishing Platform), New Delhi, India; 2 Gevanam Genomics (P) Ltd, Lucknow, Uttar Pradesh, India; 3 Biomedical Informatics (P) Ltd, Cuddalore, Tamil Nadu, India

**Keywords:** Data analytics, WoS, Scopus, freedom of expression, impact factor, India

## Abstract

Scholarly communication of data is important for advancement of science. This is done using scholarly journal platforms promoted and managed by publishers. Further, many of these journals are indexed in commercial databases such as WoS from Clarivate Inc. (Canada), SCOPUS from Elsevier Inc. (Netherlands) and several others. This inclusion is selective based on criteria defined by Clarivate Inc. and Elsevier Inc. in their capacity as private business entities. Moreover, inclusion is claimed to be representative by both parties. However, it is not comprehensive and they have large scale business activities in India. Furthermore, there is a huge hype among the scientific community and educational institutions for using data in these databases for selecting journals for scholarly communication. The WoS and SCOPUS databases have included about 300 Indian Journals. This accounts for about 1.4% journals in the database. Nonetheless, about 68% of these journals are closed access and it is managed by foreign publishers such as Wolter Kluwer, Springer, Elsevier, Francis and Taylor *etc.* This is a 2026 concern for technological embargo while balancing creativity and freedom of expression.

## Background:

Science for service in a society is the success story of scientists with serenity [1]. Hence, communication of science data is imperative across continents. This is done using scholarly journal platforms promoted and managed by publishers. Further, availability, accessibility and applicability of science data for Engineering and Technology are paramount for social developments. In this regard, accesses to known scientific data through open access publishing models have gained momentum in recent years. The Challenges and other linked features in promoting open access to Bioinformation literature over about 2 decades is clearly documented [2]. Analysis of science data is monitored through peer citation metrics. However, it is not a hard and fast rule for service in society.

An evaluation of the backward and forward citation coverage of 59 databases citation indices reports on databases that are suitable for backward and forward citations [3]. A multidisciplinary comparison of coverage via citations shows differences in coverage among databases [4]. One analysis tries to help select between Web of Science versus SCOPUS [5]. Strengths and weaknesses of PubMed, Scopus, Web of Science, and Google Scholar help to select between them as per requirement [6]. Another analysis shows that the median metrics in Web of Science's helps to overcome the major constraints of the journal impact factor [7]. Yet another analysis shows that PubMed has a sophisticated keyword optimization service, while both Scopus and Web of Science provide search analysis tools that can produce representative figures [8]. Discrepancies among coverage of funding information in medical journal articles in Scopus and Web of Science is a concern too [9]. The difference in citations in Web of Science, Scopus and Google Scholar is also alarming in using these databases [10]. Thus, there are several databases that are available for science data analysis. However, Wos and Scopus are highly popular across continents and particularly in India among educational institutions due to their belligerent marketing strategies. This is so much so that many educational institutions openly force faculties to publish their findings in Scopus indexed journals despite freedom of expression which is primary. Hence, there is control through coverage with some of these databases. It should be noted that SCOPUS is a product of Elsevier Inc. (Netherlands) with its sister companies RELX and LexisNexis [11]. Web of Science is a product of Clarivate Inc. which is owned by Onex Corporation (Canada) and Baring Private Equity Asia (Hong Kong) [12]. Further, Elsevier Inc. and Clarivate Inc. have large scale business operations in India and in most regions of Asia. Therefore, it is of interest to report the influence of foreign publishers in promoting scholarly journals from India to be indexed in WoS and SCOPUS with limited open access.

## WoS versus Scopus: 2026 Google AI [13]:

Web of Science (WoS) and Scopus are the two most prominent bibliographic databases used for academic research and evaluation (Table 1). While they overlap significantly, they differ in their philosophy, coverage, and the metrics they provide.

## Coverage & Inclusivity:

Scopus offers roughly 25-30% more journals than WoS. It is known for better coverage of non-English and regional literature, particularly from Asia and Latin America.

WoS is more selective, prioritizing high-impact journals. It is often considered more "prestigious" due to its stricter inclusion criteria.

## Citation Metrics:

## Impact Factor (WoS):

The most globally recognized measure of journal quality, used heavily in faculty promotions and grant evaluations.

## Cite Score (Scopus):

Uses a longer (four-year) citation window compared to JIF's two-year window and is generally more transparent in its calculation.

## Citation Counts:

Researchers typically see higher citation counts in Scopus because it indexes a larger pool of citing journals.

## User Experience & Tools:

Scopus is frequently praised for a more modern, intuitive interface and better visualization tools for author and institutional analytics.

WoS offers powerful advanced search features and unique tools like Cited Reference Search, which allows you to trace how a specific work has been cited over time.

## Regional & Format Differences:

## Conference Papers:

Scopus has more extensive coverage of conference proceedings, making it a favourite for Computer Science and Engineering researchers.

## Non-English Literature:

Scopus indexes significantly more non-English content (if English abstract is provided).

## Google AI suggestion:

Use Scopus if You are conducting a systematic review and need the most comprehensive results, or if your research is in the social sciences, humanities, or an interdisciplinary field.

Use Web of Science if you need historical data dating back several decades, or if your institution specifically requires Journal Impact Factor (JIF) for evaluations. For the most thorough results, many experts at institutions like Iowa State University and Arizona State University recommend searching both databases to ensure no critical research is missed.

## Publisher Origin Analysis:

## WoS-Indexed Journals from India:

## WoS-indexed journals promoted by foreign publishers:

Although 262 of 326 journals (80%) are registered with Indian addresses (Table 2), only 114 (35%) are run by genuinely Indian publishers (Table 3). The remaining 148 India-registered journals are operated by Indian subsidiaries of foreign multinational publishers (Wolters Kluwer, Springer Nature, SAGE, Elsevier) (Table 4). Combined with the 64 journals headquartered abroad, foreign-controlled publishing accounts for 212 of 326 (65%) of WoS-indexed journals classified as "Indian" (Table 5).

## Scopus-Indexed Journals from India:

## Scopus-indexed journals run by foreign multinationals:

Although 451 Scopus-indexed journals are listed under Indian publishers (Table 6), only 211 (47%) are run by genuinely Indian publishers (Table 7). The remaining 240 (53%) are operated by Indian subsidiaries of four foreign multinationals: Wolters Kluwer Medknow (184 journals), Sage Publications India (35), Springer India (20), and Reed Elsevier India (1). Of the 140 Open Access journals in the list, 125 (89%) belong to foreign MNC subsidiaries and genuinely Indian publishers contribute only 15 OA journals in Scopus. Among the 129 inactive/discontinued journals, 94 (73%) are from genuinely Indian publishers, indicating significantly higher attrition for domestic publishing operations (Table 8).

## Conclusion:

The WoS and SCOPUS databases have included about 300 Indian Journals. This accounts for about 1.4% journals in the database. Nonetheless, about 68% of these journals are closed access and it is managed by foreign publishers such as Wolter Kluwer, Springer, Elsevier, Francis and Taylor *etc.* This is a 2026 concern for technological embargo while balancing creativity and freedom of expression.

## Figures and Tables

**Table 1:** Core Differences at a Glance

**Features**	**Web of Science (WoS)**	**Scopus**
Primary Focus	Quality and selectivity ("Journals of Influence")	Breadth and inclusivity
Owner	Clarivate Analytics	Elsevier
Journal Count	~21,000 journals	~27,000-28,000 journals
Flagship Metric	Journal Impact Factor (JIF)	CiteScore
Historical Depth	Records back to 1900	Records back to 1970 (limited to 1788)
Disciplinary Strength	Natural sciences, medicine, engineering	Social sciences, arts, humanities, engineering

**Table 2:** Total Dataset

**Total WoS-Indexed Journals**	**326**
Total Open Access Journals	104
Total Non-OA Journals	222
Overall Open Access %	31.90%

**Table 3:** Publisher Classification (3 Tiers)

**Category**	**Journal Count**	**% of Total**	**OA Journals**	**Non-OA**	**OA %**
Genuinely Indian	114	35%	23	91	20.20%
Indian Subsidiary of Foreign MNC	148	45.40%	67	81	45.30%
Foreign (Headquartered Abroad)	64	19.60%	14	50	21.90%
TOTAL	326	100%	104	222	31.90%

**Table 4:** Foreign Publishers by Country (Headquartered Abroad)

**Country**	**Journal Count**	**% of Foreign**
USA	24	37.50%
England/UK	24	37.50%
Netherlands	8	12.50%
New Zealand	2	3.10%
Germany	2	3.10%
Singapore	1	1.60%
Denmark	1	1.60%
Thailand	1	1.60%
Switzerland	1	1.60%

**Table 5:** Web of Science Index Breakdown

**Index**	**Journal Count**	**% of Total**
Emerging Sources Citation Index	230	70.60%
Science Citation Index Expanded	82	25.20%
Social Sciences Citation Index	6	1.80%
Arts & Humanities Citation Index	5	1.50%
Science Citation Index Expanded | Social Sciences Citation Index	2	0.60%
Social Sciences Citation Index | Arts & Humanities Citation Index	1	0.30%

**Table 6:** Total Dataset

**Total Indian-Listed Journals**	**451**
Active Journals	322
Inactive (Discontinued) Journals	129
Total Open Access Journals	140
Total Non-OA Journals	311
Overall Open Access %	31.00%

**Table 7:** Publisher Classification (2 Tiers - All journals have Indian publisher entities)

**Category**	**Total**	**% of Total**	**Active**	**Inactive**	**OA**	**Non-OA**	**OA %**
Genuinely Indian	211	46.80%	117	94	15	196	7.10%
Indian Subsidiary of Foreign MNC	240	53.20%	205	35	125	115	52.10%
TOTAL	451	100.00%	322	129	140	311	31.00%

**Table 8:** TOP 15 Imprint Groups (Scopus parent identifier)

**Imprint Group**	**Journal Count**	**Type**	**% of Total**
Wolters Kluwer Health	184	Foreign MNC	40.80%
SAGE	35	Foreign MNC	7.80%
Springer Nature	20	Foreign MNC	4.40%
Jaypee Brothers Medical Publishers (P) Ltd	17	Indian	3.80%
Institution of Engineers (India)	16	Indian	3.50%
IP Innovative Publication Pvt. Ltd.	12	Indian	2.70%
Indian Academy of Sciences	7	Indian	1.60%
Pushpa Publishing House	7	Indian	1.60%
Research India Publications	7	Indian	1.60%
Scientific Publishers of India	7	Indian	1.60%
National Institute of Science Communication and Information Resources (NISCAIR)	5	Indian	1.10%
Innovare Academics Sciences Pvt. Ltd	4	Indian	0.90%
Indian Council of Medical Research	4	Indian	0.90%
Indian Council of Agricultural Research	3	Indian	0.70%
Indian Pharmaceutical Association	3	Indian	0.70%
